# NK1.1 Expression Defines a Population of CD4^+^ Effector T Cells Displaying Th1 and Tfh Cell Properties That Support Early Antibody Production During *Plasmodium yoelii* Infection

**DOI:** 10.3389/fimmu.2018.02277

**Published:** 2018-10-15

**Authors:** Daniel J. Wikenheiser, Susie L. Brown, Juhyung Lee, Jason S. Stumhofer

**Affiliations:** Department of Microbiology and Immunology, University of Arkansas for Medical Sciences, Little Rock, AR, United States

**Keywords:** malaria, T follicular helper cell, antibody, NK1.1, plasmablasts

## Abstract

Early plasmablast induction is a hallmark of *Plasmodium* infection and is thought to contribute to the control of acute parasite burden. Although long understood to be a T-cell dependent phenomenon, regulation of early plasmablast differentiation, however, is poorly understood. Here, we identify a population of CD4^+^ T cells that express the innate NK cell marker NK1.1 as an important source of T cell help for early plasmablast and parasite-specific Ab production. Interestingly, NK1.1^+^ CD4^+^ T cells arise from conventional, naive NK1.1^−^ CD4^+^ T cells, and their generation is independent of CD1d but critically reliant on MHC-II. CD4^+^ T cells that express NK1.1 early after activation produce IFN-γ and IL-21, and express the follicular helper T (Tfh) cell markers ICOS, PD-1 and CXCR5 more frequently than NK1.1^−^ CD4^+^ T cells. Further analysis of this population revealed that NK1.1^+^ Tfh-like cells were more regularly complexed with plasmablasts than NK1.1^−^ Tfh-like cells. Ultimately, depletion of NK1.1^+^ cells impaired class-switched parasite-specific antibody production during early *Plasmodium yoelii* infection. Together, these data suggest that expression of NK1.1 defines a population of rapidly expanding effector CD4^+^ T cells that specifically promote plasmablast induction during *Plasmodium* infection and represent a subset of T cells whose modulation could promote effective vaccine design.

## Introduction

Despite decades of research, a highly efficacious vaccine against the protozoan parasite *Plasmodium* has yet to be developed, and malaria continues to remain a significant global health problem (1). Although resistance from severe disease is mediated in part by parasite-specific Abs, protective anti-*Plasmodium* Abs are slow to develop in humans and challenging to induce artificially (2). Moreover, a clear understanding of why Ab-mediated immunity is slow to develop is still lacking. Vaccine failure has been attributed to antigenic variation and genetic polymorphisms within the *P. falciparum* (the predominant disease-causing parasite of humans) genome as a whole, as well the parasite's ability to modulate expression of essential parasite proteins such as PfEMP-1 (3). These factors, as well as others employed by the parasite, lend credence to the idea that *P. falciparum* subverts B cell responses in a manner that results in the inefficient acquisition of protective Abs (2). Thus, further insight into how *Plasmodium* infection shapes the subsequent immune response, including its impact on T and B cell differentiation, could lead to novel vaccine strategies designed to stimulate the production of high affinity, parasite-specific Abs.

Recently, glycolipid-reactive CD4^+^ NKT cells were evaluated in numerous vaccine platforms (including anti-malarial strategies such as irradiated sporozoite vaccination) due to their adjuvant potential (4, 5). NKT cells are a distinct T cell subset that express NK cell markers, intermediate levels of αβ-TCRs, and a biased repertoire of Vα and Vβ chain genes that bind lipid antigens presented in the context of the MHC class-I like molecule CD1d (abundantly expressed on professional APCs such as B cells and dendritic cells). The adjuvant potential of NKT cells is primarily based on their ability to rapidly respond to antigenic stimulation by secreting IL-4 and IFN-γ, which results in the activation of numerous immune cells, including dendritic cells, NK cells, B cells and CD4^+^ and CD8^+^ T cells (5–7).

In the context of malaria, many sporozoite and merozoite surface-localized proteins are GPI anchored. *P. falciparum* GPI can be loaded and presented on CD1d *in vitro*, resulting in the activation, proliferation, and production of IL-4 by NKT cells (8). However, the role of NKT cells in the immune response against *Plasmodium in vivo* is controversial, particularly with regard to blood stage infection. For example, CD1d-deficient mice mount a diminished Ab response during blood-stage *P. berghei* ANKA infection (9), but no difference in parasitemia or survival was noted in *Cd1d*^−/−^ mice infected with *P. yoelii* or *P.c. adami* (10, 11). Nevertheless, the identification of CD1d-independent NKT cells (7, 12) suggests subsets of conventional MHC-restricted T cells may also adopt NK-like characteristics, and potentially participate in anti-malarial immunity. For example, CD1d-independent innate-like CD8^+^ T cells were recently identified (13, 14). Furthermore, innate NK-like phenotypic characteristics were just observed in B cell subsets (14). As a whole, these studies suggest a variety of adaptive immune cells can adopt innate NK-like characteristics to accelerate, modify, or regulate conventional adaptive immunity.

Thus, as an alternative means to promote or enhance Ab production, we sought to assess the role of non-conventional, innate-like CD4^+^ T cells in the humoral response during murine *Plasmodium* infection. Here, we describe a population of CD1d-independent MHC-II-restricted NK1.1-expressing CD4^+^ TCRβ^hi^ T cells that expand dramatically during acute *P. yoelii* infection. NK1.1-expressing CD4^+^ T cells produced IFN-γ and IL-21 more abundantly than their NK1.1^−^ counterparts. Interestingly, this population showed a higher frequency of ICOS, PD-1, CXCR5 and Bcl6 expression—markers associated with Tfh cell differentiation—than non-NK1.1–expressing CD4^+^ T cells. Thus, NK1.1-expressing CD4^+^ T cells constituted a significant proportion of the early Tfh-like cell response. Strikingly, these Tfh-like NK1.1^+^ cells were found complexed with plasmablasts more frequently than their NK1.1^−^ counterparts.

Depletion of NK1.1-expressing T cells led to dramatically fewer class-switched parasite-specific Ab-secreting cells during the first week of infection, and a concomitant decrease in serum MSP-1_19_—specific Abs. Thus, infection-induced NK1.1^+^ T cells represent a T cell population critically poised to provide B cell help and promote rapid Ab production during *Plasmodium* infection.

## Materials and methods

### Mice

C57BL/6J, *Cd1d1-d2*^−/−^, *H2dIAb1-Ea*^−/−^, *Cd19*^*tm*1(*cre*)*Cgn*^ *Igh*^*b*^, B6.SJL-Ptprca Pepcb/BoyJ, and *Ighm*^*tm*1*Cgn*^ (μMT) mice were obtained from The Jackson Laboratory. Male BALB/c mice were purchased from Charles River Laboratories. Female mice between the ages of 7–9 weeks were used for all experimental cohorts. Experimental results were confirmed in similarly aged male mice to ensure that the experimental results displayed no sexual bias. Male BALB/c mice were used for routine passage of parasites, as previously described (15). All mice were housed and bred in specific-pathogen-free facilities at the University of Arkansas for Medical Sciences in accordance with institutional guidelines. The IACUC at the University of Arkansas for Medical Sciences approved all procedures on mice in this study. All animal procedures were performed in compliance with the Animal Welfare Act and accordance with the principles set forth in the “Guide for the Care and Use of Laboratory Animals,” Institute of Laboratory Animals Resources, National Research Council, National Academy Press, 2011.

### Parasites

*Plasmodium yoelii* 17XNL and *Plasmodium chabaudi chabaudi* AS infections were performed as previously described (15, 16). Briefly, cryopreserved parasite stocks were intraperitoneally (i.p.) inoculated into male BALB/c mice; at peripheral blood parasitemia of approximately 1–2%, mice were sacrificed, and blood was collected into heparinized RPMI. 10^5^ parasitized erythrocytes were subsequently i.p. injected into experimental mice to establish infection. All procedures involving *Plasmodium yoelii* 17XNL and *Plasmodium chabaudi chabaudi* AS were approved by the IBC at the University of Arkansas for Medical Sciences. All procedures were performed in compliance with the guidelines outlined in the 5th edition of “*Biosafety in Microbiological & Biomedical Laboratories*,” U.S. Department of Health and Human Services, 2009.

### Flow cytometry

Single cell suspensions were obtained by passing spleens through 40-micron filters; following ammonium chloride erythrocyte lysis; cells were re-suspended in complete RPMI (RPMI 1640 supplemented with 10% FBS, 1% non-essential amino acids, 1% sodium pyruvate, 1% L-glutamate, 1% penicillin-streptomycin, and 0.1% β-mercaptoethanol). Before FACs staining, Fc receptors on splenocytes were blocked with normal rat and mouse serum, and 2.4G2 Abs. For surface staining, Ab cocktails were re-suspended in FACs staining buffer containing 0.2% BSA and 0.2% 0.5M EDTA in PBS, followed by fixation in 4% paraformaldehyde (PFA). For intracellular cytokine staining, splenocytes were first stimulated for 4 h at 37°C in the presence of PMA, ionomycin and Brefeldin A. Cells were then surface stained and fixed with 4% PFA. Intracellular staining was performed following permeabilization with 0.1% saponin dissolved in FACs buffer. For nuclear transcription factor and Ki-67 staining, cells were first surface stained as described, followed by simultaneous fixation and permeabilization with a FoxP3 fix/perm kit per manufacturers direction (ThermoFisher Scientific). Annexin V staining was performed according to the manufacturer's instructions in the Annexin V-FITC apoptosis detection kit (ThermoFisher Scientific). To assess T-B cell interactions, FACs staining was performed as described earlier with the exception that EDTA was withheld from all staining buffers to prevent disruption of cell-cell interactions (16).

Fluorescently labeled Abs were purchased from BioLegend, ThermoFisher Scientific, or BD Biosciences. Abs used for flow cytometry include CD4, TCRβ, NK1.1, PD-1, CXCR5, CD44, CD62L, ICOS, CD8α, Annexin V, Ki-67, Ly6C, T-bet, Bcl6, Gata3, RORγt, Foxp3, IFN-γ, TNF, IL-17, CD11a, CD138, B220, CD45.2, PD-L1, IgM, CD45, Ter119, CD11c, CD11b. Fixable viability dye and propidium iodide were from ThermoFisher Scientific. For IL-21 staining, recombinant mouse IL-21 receptor fused to human Fc (R & D Systems) staining was performed first, followed by secondary anti-human Fc-PE Ab (ThermoFisher Scientific) staining. The PBS-57 analog of α-galactosylceramide loaded CD1d tetramer and unloaded CD1d negative control were acquired from the NIH Tetramer Core Facility at Emory University. Samples were acquired on a BD Biosciences LSRFortessa and analyzed using FlowJo version X software.

### ELISA

Relative parasite-specific Ab titer was assayed by coating Immulon HBX 4x plates (Thermo Scientific) with *P. yoelii* MSP-1_19_ recombinant protein or whole parasite lysate. Serially diluted serum was applied followed by incubation with HRP-conjugated IgM, and IgG-specific Abs (Southern Biotech). ELISAs were developed with SureBlue substrate (KPL), and the absorbance of each well was measured at 450 nm on a FLUOstar Omega plate reader (BMG Labtech).

### Parasitemia

Peripheral blood parasitemia was assessed by flow cytometry, as recently described (17). Briefly, ~1 uL of blood was obtained by tail vein puncture and immediately re-suspended in heparinized PBS. RBCs were washed with FACs buffer (described above) and stained with Hoechst 34580 (Life Technologies) to detect DNA, APC-labeled CD45 (to exclude lymphocytes), and PerCp-Cy5–labeled Ter119 (to label erythrocytes). Cells were obtained on a BD Biosciences LSRFortessa, and parasitemia was assessed with FlowJo version X software.

### Adoptive transfer

Naïve (CD44^lo^CD62L^hi^) CD4^+^ T cells sorted from C57BL/6J mice were transferred i.v. through retro-orbital injection into congenic CD45.1 (B6.SJL-Ptprca Pepcb/BoyJ) mice. On the following day, recipient mice were infected i.p. with 10^5^ *P. yoelii* infected RBCs. Transferred cells were recovered from infected mice on day 7 and 11 p.i. utilizing anti-CD45.2-PE, anti-PE microbeads (Miltenyi Biotech) and positive selection on an autoMACs Pro Separator (Miltenyi Biotech).

### Antibody depletion

For depletion of NK1.1-expressing cells, 200 μg of anti-NK1.1 (clone PK136, BioXcell) or isotype control mouse IgG2a (BioXcell) was i.p. injected every other day, beginning on day−1 of *P. yoelii* infection.

### Statistical analysis

Statistical analysis was performed using GraphPad Prism 7.0 (GraphPad Software, Inc., San Diego, CA). Specific tests of statistical significance are detailed in the figure legends.

## Results

### NK1.1^+^CD4^+^ T cells expand dramatically during acute *P. yoelii* infection

**Figure 1:**
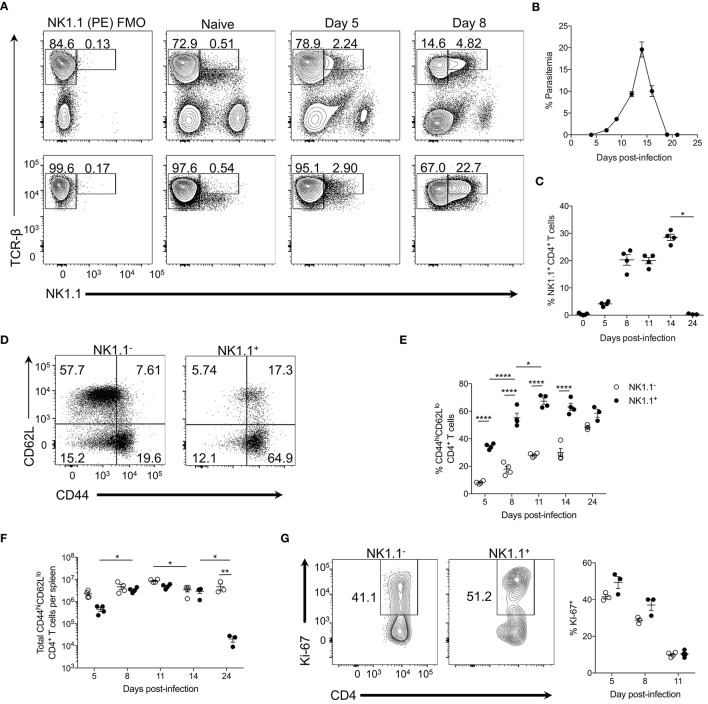
NK1.1^+^ CD4^+^ T cells expand during acute *P. yoelii* infection. **(A)** Top: Representative TCRβ and NK1.1 expression at days 5 and 8 post-*P. yoelii* infection. Gated on live splenocytes. Bottom: Representative plots that were previously gated on live, CD4^+^TCRβ^+^ splenocytes showing TCRβ and NK1.1 expression at days 5 and 8 post-*P. yoelii* infection. FMO, fluorescence minus one staining control. **(B)** Peripheral blood parasitemia as determined by FACs. **(C)** Frequency of NK1.1 expression among live CD4^+^TCRβ^+^ T cells. Significance assessed by one-way ANOVA Kruskal-Wallis test with *post-hoc* Dunn's multiple comparisons test. ^*^p < 0.05. **(D)** Representative CD44 and CD62L expression among live, NK1.1 positive and negative CD4^+^TCRβ^+^ splenocytes at day 8 post-infection. **(E)** Frequency of CD44^hi^CD62L^lo^ of live CD4^+^TCRβ^+^ T cells between days 5 and 24 post-infection. An aligned rank transformation was performed on non-parametric data before determining significance by two-way ANOVA with a *post hoc* Holm-Sidak's multiple comparisons test. ^*^p < 0.05, ^****^p < 0.0001. **(F)** Total number of NK1.1^−^ or NK1.1^+^ CD4^+^TCRβ^+^ T cells per spleen. An aligned rank transformation was performed on non-parametric data before determining significance by two-way ANOVA with a *post hoc* Holm-Sidak's multiple comparisons test. ^*^p < 0.05, ^**^p < 0.01. **(G)** Representative Ki-67 staining and frequency of expression among live, NK1.1 positive and negative T cells at day 8 post-infection. Cells previously gated on live, CD44^hi^CD62L^lo^CD4^+^TCRβ^+^ splenocytes. Data are representative of two **(G)** or three **(A–F)** independent experiments (error bars, s.e.m.).

To identify NKT cells during infection of wild-type (WT) mice with *P. yoelii* 17XNL—a non-lethal, self-resolving rodent model of malaria—splenocytes were stained for CD4, TCR-β, and NK1.1. Although a distinct population of NKT cells (CD4^+^ TCR-β^int^NK1.1^+^) was present in the spleen of naïve WT mice, this population did not expand during infection. Instead, a TCR-β^hi^NK1.1^+^ population of CD4^+^ T cells emerged in the spleen as early as day 5 post infection (p.i.) (Figure 1A), and this population phenotypically resembled NK1.1-expressing T cells previously described in the spleen following infection with *P. yoelii* sporozoites (10). Similarly, TCR-β^hi^NK1.1^+^ CD4^+^ T cells expanded in the spleen after *P. chabaudi* infection (Supplemental Figure 1A), a rodent model characterized by persistent low-level parasitemia. Additionally, NK1.1 expression was not limited to CD4^+^ T cells, as splenic CD8^+^ T cells also upregulated this marker after *P. yoelii* infection (Supplemental Figures 1B,C).

TCR-β^hi^NK1.1^+^ CD4^+^ T cells expanded rapidly and remained elevated until peak parasitemia (day 14 p.i.) but contracted significantly upon resolution of the infection at day 24 p.i. (Figures 1B,C). TCR-β^hi^NK1.1^+^ CD4^+^ T cells also contracted during the chronic stage of *P. chabaudi* infection (Supplemental Figure 1A). By day 8 p.i. the majority of the TCR-β^hi^NK1.1^+^CD4^+^ T cells exhibited a CD44^hi^CD62L^lo^ phenotype indicative of activated effector T cells (Figure 1D) and maintained this phenotype over the course of infection (Figure 1E). Not surprisingly, a significantly smaller percentage of NK1.1^−^CD4^+^ T cells displayed a CD44^hi^CD62L^lo^ phenotype during the active infection (Figure 1E), as the majority of these cells maintain a naïve phenotype. Also, TCR-β^hi^NK1.1^+^CD4^+^ T cells showed a significantly higher fold expansion in total cell numbers between day 5 and 8 p.i. compared to NK1.1^−^ CD4^+^ T cells. By day 8 p.i., similar numbers of activated (CD44^hi^CD62L^lo^) NK1.1^−^ and NK1.1^+^ CD4^+^ T cells were seen over the course of the infection except for day 24 when a significant reduction in NK1.1^+^ cell numbers occurred with no corresponding loss of NK1.1^−^ cells (Figure 1F). As TCR-β^hi^NK1.1^+^CD4^+^ T cells expanded more rapidly than NK1.1^−^CD4^+^ T cells, we sought to more closely assess their proliferation. At day 5 and 8 post-*P. yoelii* infection, we observed a higher frequency of Ki-67 expression (a nuclear antigen associated with cell cycle progression) in activated (CD44^hi^CD62L^lo^) TCR-β^hi^NK1.1^+^CD4^+^ T cells relative to NK1.1^−^CD4^+^ T cells (Figure 1G). Collectively, these data indicate that TCR-β^hi^NK1.1^+^CD4^+^ T cells represent a population of highly activated and proliferating effector CD4^+^ T cells that arise in the spleen during acute *P. yoelii* infection.

### NK1.1^+^CD4^+^ T cells are MHC-II-restricted and CD1d-independent

**Figure 2:**
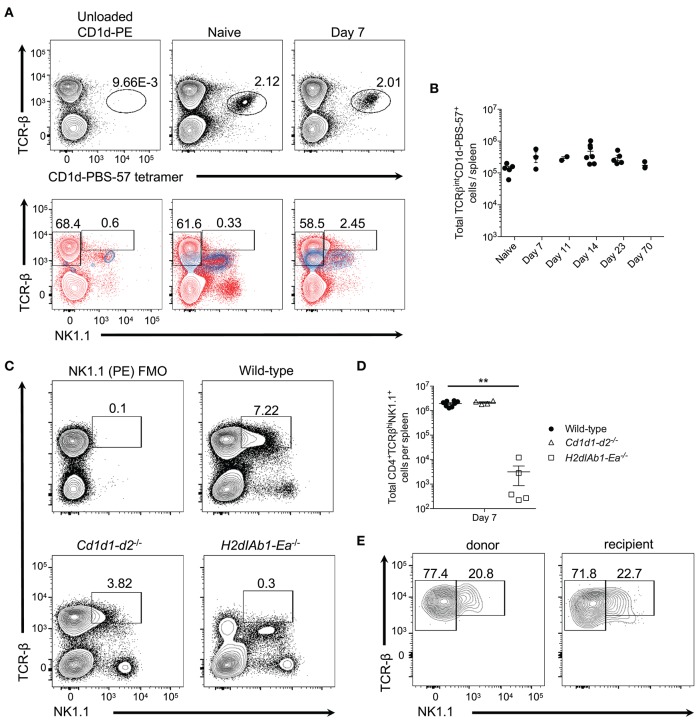
Type I invariant NK-T cells do not expand during *P. yoelii* infection. **(A)** Top: Representative TCRβ and PBS-57 tetramer staining in naive and day 7 post-*P. yoelii* infected mice. Unloaded CD1d tetramer, negative staining control. Bottom: Representative TCRβ and NK1.1 expression (blue contour plots) on unloaded CD1d tetramer^+^ cells (left), or CD1d-PBS-57 tetramer^+^ cells from naïve (middle) and day 7 infected mice (right). Red contour plots represent TCRβ and NK1.1 expression on total splenocytes. Cells previously gated on live, dump- splenocytes. **(B)** Total number of live TCRβ^int^CD1d-PBS57^+^ type I NK-T cells per spleen. Data are representative of two independent experiments (error bars, s.e.m.). **(C)** Representative TCRβ and NK1.1 expression at day 7 post-*P. yoelii* infection in wild-type, CD1d deficient (*Cd1d1-d2*^−/−^), or MHC-II deficient (*H2dIAb1-Ea*^−/−^) mice. FMO, fluorescence minus one staining control. **(D)** Total number of live CD4^+^TCRβ^+^NK1.1^+^ T cells per spleen at day 7 post-infection. Significance calculated by one-way ANOVA Kruskal-Wallis test with *post hoc* Dunn's multiple comparisons test. ^**^p < 0.01. **(E)** Representative TCRβ and NK1.1 expression on endogenous CD45.1^+^ or donor CD45.2^+^ CD4^+^ T cells at day 8 post-infection. Naïve (CD44^lo^CD62L^hi^) CD4^+^ T cells from C57BL/6 mice (CD45.2^+^) were sorted and transferred i.v. into congenic CD45.1^+^ mice 1 day before challenge with *P. yoelii*. Data are representative of two independent experiments (error bars, s.e.m.).

NK1.1 expression on CD4^+^ T cells is typically utilized for the identification of NKT cells. Therefore, we sought to determine if TCR-β^hi^NK1.1^+^ CD4^+^ T cells were indeed a population of NKT cells. Type I NKT cells represent the most well-classified subset of NKT cells and express the invariant Vα14-Jα18 TCR-α chain complexed to either a Vβ8.2, Vβ7, or Vβ2 TCR-β chain in mice, facilitating binding to glycolipid antigens displayed by the major histocompatibility (MHC) class I–like molecule CD1d (7). To assess the expansion of type I NKT cells in the spleen after *P. yoelii* infection, PBS-57-loaded (an analog of the CD1d binding glycolipid α-galactosylceramide) CD1d-tetramer was used. Although tetramer-binding TCRβ^+^ NKT cells are identifiable in the spleen after infection (Figure 2A), no significant increase in type I NKT cell numbers occurred throughout the infection (Figure 2B). Further examination of the tetramer-positive cells revealed that these cells are a heterogeneous population of TCR-β^int^ NK1.1^−^ and NK1.1^+^ NKT cells and not the TCR-β^hi^NK1.1^+^ CD4^+^ T cells that emerge after infection (Figure 2A). These data indicate that type I invariant NKT cells do not account for the increase in NK1.1-expressing CD4^+^ T cells observed during acute *P. yoelii* infection.

While CD1d tetramers are capable of identifying type I NKT cells, type II NKT cells are not as easily detected, despite similar CD1d restriction (7). Thus, to determine if the NK1.1^+^CD4^+^ T cells observed here are type II NKT cells, WT, CD1d-deficient (lacking type I and II NK-T cells), and MHC-II-deficient (lacking conventional CD4^+^ T cells) mice were infected with *P. yoelii*. As expected based on previous findings (10, 18) class II-deficient mice were unable to control peak parasitemia, while *Cd1d1-d2*^−/−^ mice showed higher parasitemia that peaked on day 18 compared to day 16 in WT mice, but they resolved the infection within the same time frame as WT mice (Supplemental Figure 2A). Interestingly, the absence of CD1d did not impair the expansion of TCR-β^hi^NK1.1^+^ CD4^+^ T cells, but the lack of MHC-II expression ablated induction of this subset (Figures 2C,D). This data indicates that NK1.1-expressing T cells are derived from a population of CD4^+^ T cells that bind protein antigen presented in the context of MHC-II. Importantly, NKT cells (TCR-β^int^CD4^+^ NK1.1^+^) are present in all three genotypes of mice following infection, including *Cd1d1-d2*^−/−^ mice; this latter population most likely represents previously described CD1d-independent NKT cells (7). To demonstrate that TCR-β^hi^NK1.1^+^ T cells are derived from conventional CD4^+^ T cells that do not express NK1.1 naïve NK1.1^−^CD4^+^ T cells were sorted from uninfected donor mice and transferred into congenic mice followed by infection of recipient mice with *P. yoelii* 1 day after transfer. Activated donor and endogenous CD4^+^ T cells expressed NK1.1 at a similar frequency on day 7 p.i., indicating that TCR-β^hi^NK1.1^+^CD4^+^ T cells are indeed derived from NK1.1^−^CD4^+^ T cells (Figure 2E).

To further alleviate the concern that the observed NK1.1 staining was an artifact of flow cytometry staining (i.e., non-specific labeling), we also assessed the induction of TCR-β^hi^NK1.1^+^CD4^+^ T cells in *P. yoelii*-infected BALB/c mice, which do not express the protein NK1.1. As expected, unlike in C57BL/6 mice, we did not identify TCR-β^hi^NK1.1^+^CD4^+^ T cells after *P. yoelii* infection in BALB/c mice. Additionally, we did not observe staining with the isotype control Ab IgG2a in C57BL/6 or BALB/c mice, further validating the accuracy of NK1.1 staining (Supplemental Figure 3). Together, these data suggest that infection-induced NK1.1-expressing CD4^+^ T cells are not type I or II NKT cells but are instead a subset of conventional MHC-II-restricted CD4^+^ T cells, hereafter referred to as NK1.1^+^ CD4^+^ T cells.

### NK1.1^+^CD4^+^ T cells express transcription factors associated with the Th1 and Tfh cell lineage that are poised to produce cytokines related to these subsets

**Figure 3:**
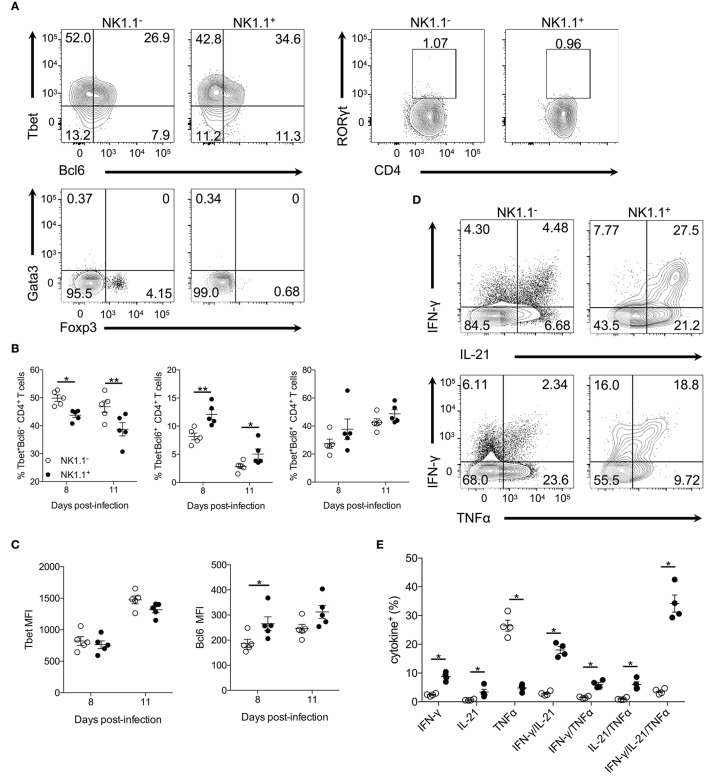
NK1.1^+^CD4^+^ T cells express Tbet and Bcl6 and produce effector cytokines. **(A)** Representative intracellular staining of NK1.1^−^ and NK1.1^+^ CD4^+^ T cells for the transcription factors Tbet, Bcl6, RORγt, Gata3, and Foxp3. Cells previously gated on live, CD44^hi^CD62L^lo^CD4^+^TCRβ^+^ splenocytes. Gates based on FMO controls shown in Supplemental Figure 4. **(B)** Frequency of NK1.1^+^ and NK1.1^−^ CD4^+^ T cells expressing Tbet, Bcl6 or, Tbet and Bcl6 on days 8 and 11 p.i. An aligned rank transformation was performed on non-parametric data before determining significance by two-way ANOVA with a *post hoc* Holm-Sidak's multiple comparisons test. *p < 0.05, **p < 0.01. **(C)** Median fluorescence intensity (MFI) for Tbet and Bcl6 in NK1.1^+^ and NK1.1^−^ CD4^+^ T cells on day 8 and 11 p.i. An aligned rank transformation was performed on non-parametric data before determining significance by two-way ANOVA with a *post hoc* Holm-Sidak's multiple comparisons test. *p < 0.05. **(D)** Representative intracellular cytokine staining of NK1.1^−^ and NK1.1^+^ CD4^+^ T cells previously gated on live CD4^+^TCRβ^+^ T cells, on day 7 post-infection. Splenocytes were stimulated for 4 h with PMA and ionomycin in the presence of brefeldin A before being stained for IFN-γ, IL-21, and TNFα. **(E)** The frequency of single, double, or triple cytokine-producing cells at day 7 post-infection. Significance assessed via an unpaired non-parametric Mann-Whitney test. *p < 0.05. Data are representative of three independent experiments (error bars, s.e.m.).

To determine if NK1.1^+^ CD4^+^ T cells differ phenotypically from their NK1.1^−^ counterparts we first assessed their ability to express T-bet and Bcl6, transcription factors associated with T helper type 1 (Th1) and follicular helper T (Tfh) cell differentiation respectfully. Four primary populations emerged from this analysis: Tbet^+^Bcl6^−^, Tbet^−^Bcl6^+^, Tbet^+^Bcl6^+^, and T cells that did not express either Tbet or Bcl6, all of which were apparent at day 8 and 11 p.i. (Figure 3A, Supplemental Figure 4 and data not shown). Although all four of these populations existed within the activated (CD44 hiCD62L^lo^) NK1.1^+^ and NK1.1^−^ pools, a significantly higher percentage of the NK1.1^−^ T cells expressed Tbet at day 8 and 11 p.i., while the frequency of Bcl6 expression amongst NK1.1^+^ T cells was significantly higher at day 8 and 11 p.i. No difference in the percentage of Tbet^+^Bcl6^+^ cells was seen between NK1.1^+^ and NK1.1^−^ T cells (Figure 3B). Furthermore, the median fluorescent intensity (MFI) for Bcl6 expression was significantly higher at day 8 p.i. in the NK1.1^+^ population, and this trend continued at day 11 p.i. (Figure 3C). Although Th1 and Tfh cells are the predominant CD4^+^ T helper cell subsets that emerge after *Plasmodium* infection (19), we also wanted to assess transcription factors associated with other T helper cell subsets, such as RORγt (Th17), Gata3 (Th2), and Foxp3 (Treg). No distinct populations of RORγt or Gata3-expressing CD4^+^ T cells were seen at day 8 p.i. within either the NK1.1^+^ or NK1.1^−^ populations. Though there was a sizable population of Foxp3^+^ regulatory T cells within the NK1.1^−^ pool this was not the case for the NK1.1-expressing population (Figure 3A and Supplemental Figure 4).

CD4^+^ T cells produce many cytokines that are essential for their effector response after *Plasmodium* infection, including IFN-γ, IL-21, and TNF-α (20–23). To determine if NK1.1^+^ T cells are capable of producing these effector cytokines splenocytes were re-stimulated directly ex vivo. Interestingly, a significantly higher percentage of NK1.1^+^CD4^+^ T cells was found to produce IFN-γ and IL-21, but less TNF-α than NK1.1^−^CD4^+^ T cells. Also, a higher frequency of NK1.1^+^CD4^+^ T cells was found to produce two (IFN-γ^+^IL-21^+^, IFN-γ^+^TNF-α^+^, IL-21^+^TNF-α^+^) or all three (IFN-γ^+^IL-21^+^TNF-α^+^) cytokines than NK1.1^−^CD4^+^ T cells (Figures 3D,E). These data suggest NK1.1^+^CD4^+^ T cells share similarities with activated NK1.1^−^CD4^+^ T cells. Also, the finding that a significant proportion of NK1.1^+^CD4^+^ T cells produce pro-inflammatory cytokines fits with their pronounced effector phenotype (Figure 1). Together, these data indicate the expression of NK1.1 does not prevent or limit differentiation of these CD4^+^ T cells into a particular T helper subset, as NK1.1^+^CD4^+^ T cells display characteristics of Th1 and Tfh cells. Although, it appears these cells slightly favor Tfh over Th1 cell differentiation compared to NK1.1^−^ T cells.

### NK1.1^+^CD4^+^ T cells are predominantly Tfh-like cells

**Figure 4:**
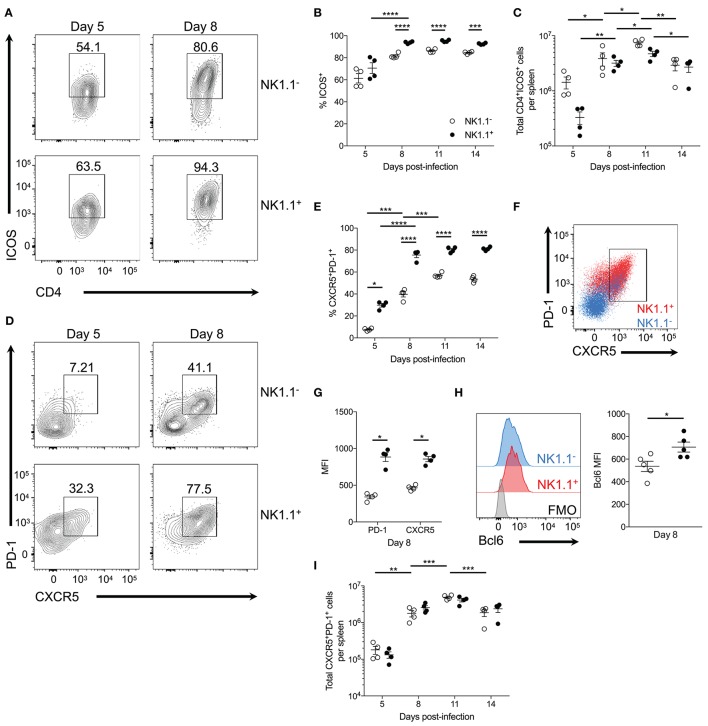
NK1.1^+^CD4^+^ T cells are primarily Tfh-like cells. **(A)** Representative ICOS expression among live activated (CD44^hi^CD62L^lo^) NK1.1 positive and negative CD4^+^TCRβ^+^ T cells at days 5 and 8 post-infection. **(B)** Frequency of ICOS expression among live activated NK1.1 negative or positive CD4^+^ T cells between days 5 and 14 post-infection. **(C)** Total number of live activated NK1.1^−^ or NK1.1^+^ ICOS^+^CD4^+^TCRβ^+^ T cells per spleen. **(D)** Representative PD-1 and CXCR5 expression on live activated NK1.1 negative or positive CD4^+^ T cells at days 5 and 8 post-infection. **(E)** Frequency PD-1^+^CXCR5^+^ of live activated CD4^+^TCRβ^+^ T cells. **(F)** PD-1 and CXCR5 expression overlay of NK1.1^+^ (red) or NK1.1^−^ (blue) CD4^+^TCRβ^+^ T cells at day 8 post-infection. **(G)** MFI of PD-1 and CXCR5 expression. **(H)** Intracellular Bcl6 staining of live activated NK1.1^+^ or NK1.1^−^ CXCR5^+^PD-1^+^ cells at day 8 post-infection and MFI of Bcl6 expression. **(I)** Total number of live activated NK1.1 positive or negative CXCR5^+^PD-1^+^ cells at day 8 post-infection. MFI, median fluorescence intensity. **(B,C,E,I)** An aligned rank transformation was performed on non-parametric data before determining significance by two-way ANOVA with a *post hoc* Holm-Sidak's multiple comparisons test. ^*^p < 0.05, ^**^p < 0.01, ^***^p < 0.001, ^****^p < 0.0001. **(G,H)** Significance assessed via an unpaired non-parametric Mann-Whitney test. ^*^p < 0.05. Data are representative of three independent experiments (error bars, s.e.m.).

The capacity to produce IL-21, a cytokine involved in germinal center B cell reactions and the high expression of Bcl6 by NK1.1^+^CD4^+^ T cells suggested that these cells might represent an early Tfh cell population. Therefore, we evaluated additional markers associated with Tfh cell differentiation on activated CD4^+^ T cells (CD44^hi^CD62L^lo^). ICOS, a co-stimulatory molecule implicated in the induction and maintenance of Tfh cells (24–26), was expressed on both NK1.1 positive and negative CD4^+^ T cells on day 5 after infection. (Figures 4A,B). By day 8 p.i. greater than 90% of the NK1.1^+^CD4^+^ T cells expressed ICOS, and the frequency of ICOS^+^ cells remained significantly higher amongst the NK1.1^+^CD4^+^ T cells through the peak of infection (Figures 4A,B). Despite a higher rate of ICOS expression amongst NK1.1^+^CD4^+^ T cells, there were more ICOS^+^ NK1.1^−^CD4^+^ T cells on day 5 p.i. By day 8 both populations of CD4^+^ T cells showed a significant expansion in ICOS^+^ cells that continued through day 11 with both populations showing a substantial reduction in ICOS expression at day 14 p.i. However, the gap in the number of ICOS^+^ cells seen on day 5 p.i. closed by day 8 p.i., as a similar number of ICOS^+^ cells was seen within the NK1.1^−^ and NK1.1^+^ populations and numbers remained comparable through day 14 (Figure 4C).

As ICOS signaling is implicated in Tfh cell priming, expression of PD-1 and CXCR5—lineage-defining Tfh cell proteins—was assessed. At day 5 p.i. a significant proportion of NK1.1^+^CD4^+^ T cells already expressed PD-1 and CXCR5 compared to NK1.1^−^CD4^+^ T cells. Although the expression of PD-1 and CXCR5 significantly increased amongst both NK1.1 positive and negative CD4^+^ T cells between days 5 and 8 p.i., NK1.1^+^CD4^+^ T cells more frequently expressed PD-1 and CXCR5 relative to NK1.1^−^CD4^+^ T cells throughout the first two weeks of infection (Figures 4D,E). Also, a higher proportion of NK1.1^+^CD4^+^ T cells expressed CXCR5 and PD-1 after *P. chabaudi* infection than conventional CD4^+^ T cells (Supplemental Figure 1). Moreover, superimposed plots of PD-1 and CXCR5 expression indicated that NK1.1^+^ T cells expressed significantly more PD-1 and CXCR5 on a cell to cell basis relative to NK1.1^−^ T cells, as shown by MFI at day 8 p.i. (Figures 4F,G). In support of the data in Figure 3, Bcl6 expression was detected in both NK1.1 positive and negative PD-1^+^CXCR5^+^ cells, but the expression of Bcl6 was significantly higher in NK1.1^+^ T cells (Figure 4H). Hence, the expression of ICOS, PD-1, CXCR5 and Bcl6 by NK1.1^+^ and NK1.1^−^ CD4^+^ T cells defines these cells as Tfh-like cells. Ultimately, the total number of NK1.1^+^ and NK1.1^−^ Tfh-like cells did not differ dramatically over the course of *P. yoelii* infection, although both populations showed a significant expansion in cell numbers from day 5–8 and day 8–11 p.i., and they showed a significant reduction in numbers from day 11–14 p.i. (Figure 4I). Together, these data indicate NK1.1^+^CD4^+^ T cells are primed to preferentially adopt a Tfh cell phenotype, although a proportion of the cells are capable of expressing features associated with Th1 cells (Tbet and IFN-γ), suggesting that CD4^+^ T cells, in general, are not fully committed to a Tfh cell fate at this time after infection. Furthermore, an increase in the expression of PD-1 and CXCR5 suggests that the Tfh-like NK1.1^+^ cells have a distinct, unique identity within the total Tfh cell pool.

### NK1.1^+^ Tfh-like cells interact with plasmablasts

**Figure 5:**
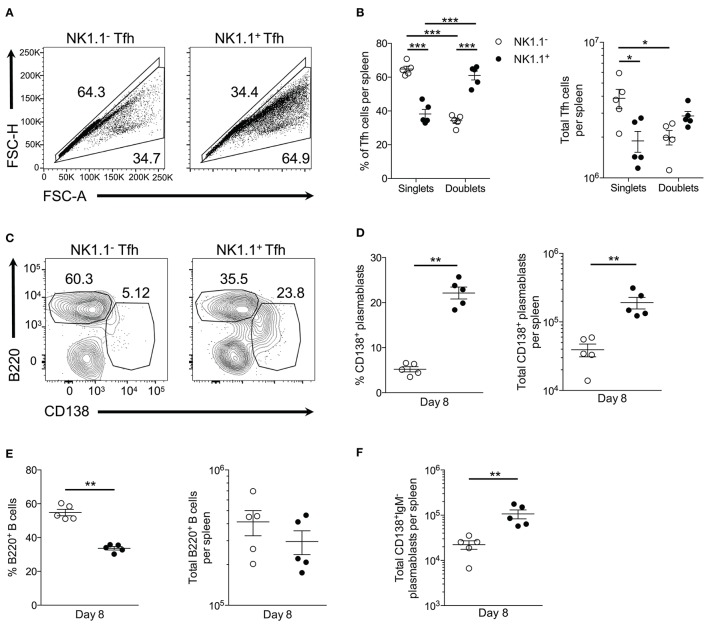
NK1.1^+^ Tfh-like cells frequently complex with plasmablasts. **(A)** Gating strategy for doublet (FSC-A^high^FSC-H^high^) and singlet (FSC-A^low^FSC-H^low^) live NK1.1 positive or negative Tfh-like (CXCR5^+^PD-1^+^) cells at day 8 post-infection. **(B)** Frequency and the total number of live singlet and doublet NK1.1 positive or negative Tfh-like cells per spleen at day 8 post-infection. An aligned rank transformation was performed on non-parametric data before determining significance by two-way ANOVA with a *post hoc* Holm-Sidak's multiple comparisons test. ^*^p < 0.05, ^***^p < 0.001. **(C)** Representative B220 and CD138 staining on live doublet NK1.1 negative or positive Tfh-like cells at day 8 post-infection. **(D)** Frequency and the total number of live CD138^+^B220^lo^ plasmablasts interacting with NK1.1 negative or positive Tfh-like cells per spleen. **(E)** Frequency and the total number of live B220^+^ B cells interacting with NK1.1 negative or positive Tfh-like cells per spleen. **(F)** Total number of live class-switched IgM^−^CD138^+^ plasmablasts interacting with NK1.1 negative or positive Tfh-like cells per spleen. **(D–F)** Significance calculated by an unpaired nonparametric Mann-Whitney test. ^**^p < 0.01. Data are representative of three independent experiments (error bars, s.e.m.).

Our observations thus far indicate NK1.1^+^CD4^+^ T cells expand rapidly following acute *P. yoelii* infection and favor the adoption of an ICOS^+^PD-1^high^CXCR5^high^ Tfh-like phenotype. As such, we sought to assess the nature of NK1.1^+^ Tfh cell help. Surprisingly, an in-depth ex vivo analysis revealed a substantial number of NK1.1^+^ Tfh-like cells complexed to or interacting with other immune cells (termed doublets, as indicated by high FSC-H and FSC-A) at day 8 p.i. Indeed, NK1.1^+^ Tfh-like cells were more likely to be found as doublets when compared to NK1.1^−^ Tfh-like cells (Figures 5A,B). Conversely, the total number of singlet NK1.1^−^ Tfh-like cells was significantly greater than doublet NK1.1^−^ Tfh-like cells, suggesting NK1.1^−^ Tfh-like cells less frequently formed stable interactions with other immune cells (Figure 5B).

We, therefore, hypothesized NK1.1^+^ Tfh-like cells promoted the T-cell dependent wave of plasmablast differentiation that is characteristic of an acute *P. yoelii* infection. Conjugate formation was assessed during early *P. yoelii* infection to determine if plasmablasts (B220^low^CD138^+^) interact with NK1.1^+^ Tfh-like cells. At day 8 p.i., a higher percentage and number of NK1.1^+^ Tfh-like cells were found complexed to plasmablasts than NK1.1^−^ Tfh-like cells (Figures 5C,D). In contrast, a significantly higher frequency of NK1.1^−^ Tfh-like cells was found complexed to B220^+^ B cells, although no significant difference in the number of NK1.1^−^ and NK1.1^+^ Tfh-like cells interacting with B cells was observed (Figure 5E). Also, the majority of the plasmablasts interacting with NK1.1^+^ Tfh-like cells were class-switched, while NK1.1^−^ Tfh-like cells interacted equally with IgM^+^ and IgM^−^ plasmablasts (Figure 5F). As a whole, these data indicate that while a similar number of NK1.1^−^ Tfh-like cells and NK1.1^+^ Tfh-like cells interact with B cells, NK1.1^+^ Tfh-like cells are more likely to be found complexed with plasmablasts.

**Figure 6:**
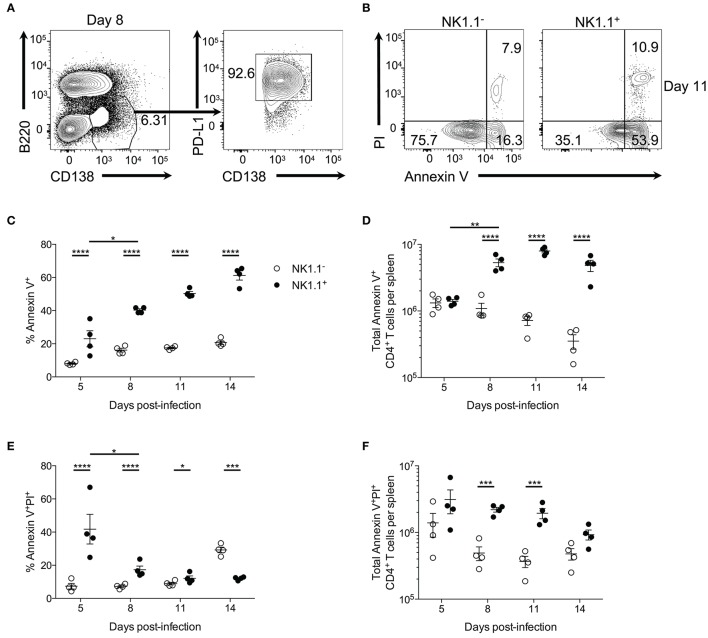
NK1.1^+^CD4^+^ T cells undergo accelerated apoptosis. **(A)** Representative B220 and CD138 expression at day 8 post-infection. PD-L1 and CD138 expression of gated area at left. **(B)** Typical PI and Annexin V staining of NK1.1 negative or positive CD4^+^TCRβ^+^ T cells at day 11 post-infection. **(C)** Frequency and **(D)** the total number of Annexin V^+^ NK1.1 negative or positive CD4^+^ T cells per spleen. **(E)** Frequency and **(F)** total number of Annexin V^+^PI^+^ NK1.1 negative or positive CD4^+^ T cells per spleen. An aligned rank transformation was performed on non-parametric data before determining significance by two-way ANOVA with a *post hoc* Holm-Sidak's multiple comparisons test. ^*^p < 0.05, ^**^p < 0.01, ^***^p < 0.001, ^****^p < 0.0001. Data are representative of two independent experiments (error bars, s.e.m.).

Further examination of plasmablasts at day 8 p.i. revealed that these cells uniformly express PD-L1 (Figure 6A). As NK1.1^+^ CD4^+^ T cells express high amounts of the PD-L1 receptor PD-1, we hypothesized these cells might be more prone to undergo apoptosis than NK1.1^−^ CD4^+^ T cells. In support of this argument, previous findings have linked PD-1 signaling to the regulation of anti-apoptotic protein expression (27–29). Propidium iodide uptake, and Annexin V expression were evaluated by flow cytometry to determine if a higher frequency of NK1.1^+^ CD4^+^ T cells are undergoing apoptosis compared to NK1.1^−^ CD4^+^ T cells. Indeed NK1.1^+^CD4^+^ T cells more frequently expressed the early apoptosis indicator Annexin V from day 5 to day 14 after infection relative to NK1.1^−^CD4^+^ T cells (Figures 6B,C). Furthermore, significantly more Annexin V^+^ NK1.1^+^CD4^+^ T cells were identified in the spleen at days 8, 11, and 14 relative to NK1.1^−^CD4^+^ T cells (Figure 6D). There was also an increase in the frequency and number of AnnexinV^+^PI^+^ NK1.1^+^CD4^+^ T cells compared with NK1.1^−^CD4^+^ T cells from day 5–11 p.i., while a higher percentage of NK1.1^−^CD4^+^ T cells were AnnexinV^+^PI^+^ at day 14 p.i. (Figures 6B,E,F). Although, the difference in total AnnexinV^+^PI^+^ cells at day 14 was not significantly different between the two populations (Figure 6F). Together, this data suggests that NK1.1^+^CD4^+^ T cells more readily undergo apoptosis relative to NK1.1^−^CD4^+^ T cells and offers one explanation as to why NK1.1^+^ T cells do not persist following resolution of acute infection.

### NK1.1^+^CD4^+^ T cells promote early plasmablast formation

**Figure 7:**
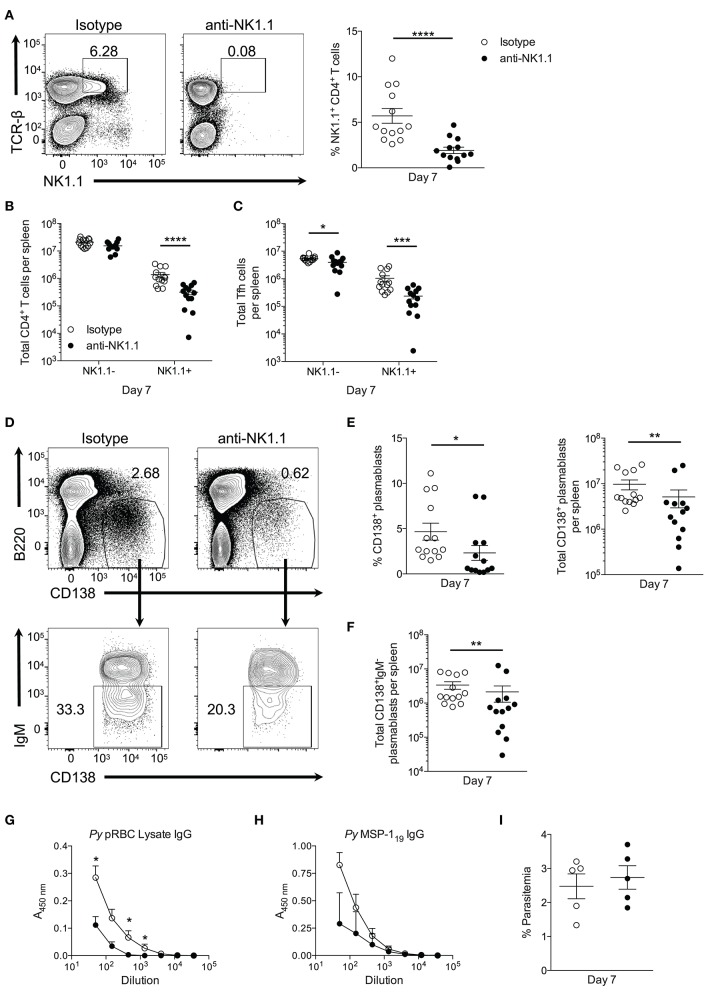
Depletion of NK1.1^+^CD4^+^ T cells inhibits parasite-specific Ab production. **(A)** The frequency of NK1.1 staining of live TCR-β^hi^NK1.1^+^ CD4^+^ T cells in isotype control or anti-NK1.1 Ab-treated mice at day 7 post-infection. **(B)** Total number of live splenic NK1.1 positive or negative CD4^+^TCRβ^hi^ T cells in isotype control or anti-NK1.treated mice at day 7 post-infection. **(C)** Total number of live splenic NK1.1 positive or negative Tfh-like (CXCR5^+^PD-1^+^) cells in isotype control or anti-NK1.1 Ab-treated mice. **(D)** Representative B220 and CD138 expression in isotype control or anti-NK1.1 Ab-treated mice at day 7 post-infection (above). Typical IgM staining of the gated area in **(D)** (below). **(E)** Frequency and the total number of live B220^low^CD138^+^ plasmablasts or **(F)** IgM^−^ class-switched plasmablasts in isotype control or anti-NK1.1 Ab-treated mice. **(A–C,E,F)** Significance determined via an unpaired nonparametric Mann-Whitney test. ^*^p < 0.05, ^**^p < 0.01, ^***^p < 0.001, ^****^p < 0.0001. **(G)** Relative pRBC-lysate specific or **(H)** MSP-1_19_-specific IgG Ab titer at day 7 post-infection in isotype control or anti-NK1.1 Ab-treated mice. Significance determined by two-way ANOVA *post hoc* Bonferroni's multiple comparisons test. ^**^p < 0.01, ^****^p < 0.0001. **(I)** Peripheral blood parasite load in isotype control or anti-NK1.1 Ab-treated mice. Data are representative of three combined experiments **(A–F)** or three independent experiments **(G–I)** (error bars, s.e.m.).

To assess the contribution of NK1.1^+^ T cells to early plasmablast differentiation, we sought to deplete this population during *P. yoelii* infection by utilizing an anti-NK1.1 depleting Ab. Treatment with anti-NK1.1 efficiently depleted NK1.1-expressing cells in most cases (Figure 7A), resulting in significantly fewer splenic TCRβ^hi^NK1.1^+^ T cells at day 7 p.i. (Figure 7B). No impact on total NK1.1^−^CD4^+^ T cell numbers was observed during administration of the anti-NK1.1 Ab. As anticipated, the depletion of NK1.1^+^CD4^+^ T cells, resulted in significantly fewer NK1.1^+^ Tfh-like cells at day 7 p.i. (Figure 7C). A decline in NK1.1^−^ Tfh-like cells was also observed at this time with anti-NK1.1 treatment.

Plasmablast differentiation was assessed at day 7 p.i. to determine if NK1.1^+^ T cells participate in early Ab production. Depletion of NK1.1-expressing cells resulted in a significant decrease in the frequency and the total number of plasmablasts at day 7 p.i. relative to isotype control Ab-treated mice (Figures 7D,E). Furthermore, substantially fewer class-switched IgM^−^ plasmablasts were observed in mice treated with anti-NK1.1 (Figures 7D,F). Assessment of serum Ab production revealed a reduction in pRBC lysate and MSP-1_19_—specific IgG Abs in NK1.1-depleted mice (Figures 7G,H). Depletion of NK1.1^+^ cells, which includes innate NK cells–a potential source of early IFN-γ-did not significantly impact parasitemia, as no measurable difference in parasite burden occurred before day 7 p.i. (Figure 7I). However, we cannot rule out that depletion of innate NK cells contributed to the observed reduction in plasmablast and Ab production with Ab treatment. We can conclude that the effects of anti-NK1.1 treatment on plasmablast and parasite-specific Ab production were not due to depletion of CD1d-dependent NK-T cells, as no difference in the output of MSP-1_19_-specific IgG between WT and *cd1d-d2*^−/−^ mice was observed (Supplemental Figure 2B). Our attempt to assess if the absence of NK1.1^+^ cells impacts parasite burden and Ab production beyond day 7 p.i. were unsuccessful, as continuous treatment with anti-NK1.1 Abs or a change in the treatment regimen to delivering the Abs every 3 days did not efficiently deplete NK1.1^+^CD4^+^ T cells long-term (*data not shown*). As a whole, these data indicate that NK1.1-expressing CD4^+^ T cells interact with B cells and plasmablasts after *P. yoelii* infection, and suggest that they help to promote early plasmablast expansion, class-switching, and Ab production, which may or may not be due to direct interactions between these cell types.

## Discussion

The precise role of CD1d-restricted NK-T cells during blood-stage *Plasmodium* infection remains controversial. Previous literature, for instance, indicates that *P. yoelii*-infected CD1d-deficient mice exhibit delayed parasite clearance (30) and harbor greater parasite burden (author's unpublished observation) relative to WT mice. Although CD1d-deficient mice mount a moderately inadequate class-switched Ab response during *P. berghei* ANKA infection (9), and others (10) have observed no significant defect in parasite-specific Ab production during blood-stage *P. yoelii* infection. Here we show that CD1d deficient mice display higher peak parasitemia but have no defect in parasite-specific Ab production (Supplemental Figure 2) or evidence of CD1d-restricted T cells assuming a Tfh cell phenotype. Furthermore, this study demonstrates infection-induced NK1.1^+^ CD4^+^ T cells are critically reliant on MHC-II antigen presentation for their development.

As a whole, NK1.1^+^CD4^+^ T cells appear to represent a CD1d-independent source of highly functional early Tfh cells. However, the phenomena of NKT-like MHC-restricted T cells are not exclusively restricted to *Plasmodium* infection. In fact, >90% of virus-specific MHC-I- and MHC-II-restricted CD8^+^ and CD4^+^ T cells express markers associated with NK cells including NK1.1 (31). Furthermore, CD1d-independent CD4^+^ and CD8^+^ NKT-like cells identified in humans preferentially expand with age (32). Similarly, NK1.1-expressing CD8^+^ CD1d-independent T cells develop in mice following LPS-primed DC immunization. In this case, NK-like CD8^+^ T cells expressed diverse TCRαβ chains and suppressed Ag-specific T cell responses (14). As the NK1.1^+^CD4^+^ T cells identified in this study are not CD1d-restricted, these cells are most likely derived from a pool of diverse TCRαβ chains.

How then are MHC-II-restricted NK1.1^+^CD4^+^ T cells categorized amongst conventional CD4^+^ T cells and NK-T cells? Our findings indicate that NK1.1^+^ and NK1.1^−^ CD4^+^ T cells are composed of a heterogeneous population of cells expressing markers associated with Th1 and Tfh cells, similar to what has been reported by others (21, 23, 33–35), further supporting the idea that T cells transition through an intermediate state prior to full commitment toward Th1 or Tfh cell differentiation (35). Perhaps, NK1.1 expression defines one of these intermediate states. The distinguishing feature of NK1.1^+^ CD4^+^ T cells is their heightened expression of ICOS, CXCR5, PD-1, and Bcl6, suggesting they favor a Tfh cell fate. However, examination of CD4^+^ T cells at a time (Day 24) when the germinal response is prevalent revealed that the GC Tfh cells (PD-1^hi^CXCR5^+^) consist of only NK1.1^−^ CD4^+^ T cells (*data not shown*). Indeed, we observed a significant contraction in the number of NK1.1-expressing CD4^+^ T cells at this time due to an increased rate of programmed cell death (Figures 1, 6). Rather than favoring their entry into the B cell follicle the early and heightened expression of proteins associated with a Tfh cell fate may instead favor interactions with B cells serving the purpose to promote and support early plasmablast differentiation.

Another factor that could influences CD4^+^ T cell effector fate determination is the length of peptide:TCR interaction (dwell time), as well as antigen dose (36, 37). As Th1 and Tfh cell differentiation is finely balanced by the ratio of Blimp-1:Bcl6, it is possible that high amounts of peptide:MHC expression by DCs during the initial DC:CD4^+^ T cell interactions primarily lead to induction of ICOS and subsequent Bcl6 expression, as well as upregulation of NK1.1. This outcome could favor the early formation of Tfh-like cells, which appear to be responsible for early T-dependent plasmablast differentiation—a key event in the control of *P. yoelii* infection. Indeed, depletion of NK1.1-expressing cells yields a dramatic defect in initial parasite-specific plasmablast production. However, as most plasmablasts are NK1.1^+^ doublets, it is likely NK1.1^+^ T cell-complexed plasmablasts are simultaneously depleted. Strong NK1.1^+^ Tfh:plasmablast interaction suggests that these NK1.1^+^ Tfh-like cells are essential for plasmablast differentiation or immediate survival. However, the extrafollicular plasmablast response is short-lived, and these non-mutated Ab-secreting cells do not persist in the spleen for an extended period, implying the majority of NK1.1^+^ Tfh-like cells are short-lived effectors. As such, the NK1.1^−^ T cells (which less frequently interacted with plasmablasts) that express Tfh cell markers may proceed into the B cell follicle to promote GC formation and continue their differentiation into GC Tfh cells.

As mentioned, NK1.1 belongs to the NKRP1 (NK receptor protein) family. Unlike killer cell Ig-like receptors (KIR), NKRP1 family members do not bind MHC class-I-like ligands. Instead, NKRP1 receptors in mice, rats, and humans have been shown to engage Clec2 (C-type lectin-like receptor 2) subfamily proteins (38). Engagement of NKRP1 receptors can lead to activation or inhibition of NK cells, although NK1.1 does not possess a consensus ITIM in its cytoplasmic domain (39), suggesting it lacks inhibitory function. NK1.1 possesses a positively charged arginine residue near its extracellular region in its transmembrane domain (40), which indicates that NK1.1 could interact with negatively charged transmembrane proteins such as the CD3 subunits on T cells to promote signaling through the TCR, which could result in enhanced activation or proliferation of T cells.

Despite these insights, the factors responsible for promoting NK1.1 expression itself remain elusive. It was previously shown that CD8^+^ T cells upregulate NK1.1 within 48–72 h in response to IL-2, IL-4, and IL-15 addition *in vitro* (13). We found that a higher percentage of NK1.1^+^ T cells displayed IL-2Rα and β expression upon their emergence at day 5 p.i. compared to NK1.1^−^CD4^+^ T cells (Supplemental Figure 5), suggesting that they preferentially respond to IL-2 or IL-15. While the purpose and function of NK1.1 expression by CD4 and CD8 T cells is still unclear, its appearance likely represents a state of activation rather than a distinct cell lineage.

Ultimately, defective parasite-specific Ab production failed to result in the acceleration of parasite burden. Therefore, how essential are NK1.1^+^ T cells in the induction of protective immunity? Ineffective depletion of NK1.1^+^ cells during late *P. yoelii* infection may partially explain why a robust protective phenotype did not emerge in this infection model. Until better tools become available for studying the function of NK1.1 on T cells, it will be difficult to understand the importance of this cell population in the immune response to *Plasmodium*. Based on the depletion studies, it is likely that this protein does not play an essential role in the immune response due to redundancy in the system. Therefore, confirmation of the localization of NK1.1^+^CD4^+^ T cells solely to the extrafollicular region in the red pulp in the spleen would facilitate the use of this protein as a marker for identifying and characterizing extrafollicular Tfh cells. Moreover, a greater understanding of the development of NK1.1^+^CD4^+^ T cells, as well as the fate of NK1.1^+^ Tfh-like cells, may help instruct future rational anti-malarial vaccine design.

## Author contributions

DW and JS designed, analyzed and interpreted the results of the study. DW, SB, and JL carried out the experiments associated with the study. DW and JS wrote the manuscript.

### Conflict of interest statement

The authors declare that the research was conducted in the absence of any commercial or financial relationships that could be construed as a potential conflict of interest.
