# Supplementary material for: NK1.1 Expression Defines a Population of CD4+ Effector T Cells Displaying Th1 and Tfh Cell Properties That Support Early Antibody Production During Plasmodium yoelii Infection
Source: Front Immunol. 2018 Oct 15;9:2277. doi: 10.3389/fimmu.2018.02277 (PMC6196288; doi:10.3389/fimmu.2018.02277)
Supplement: Supplementary file 1 [file Data_Sheet_1.PDF]

Supplemental Figure 1

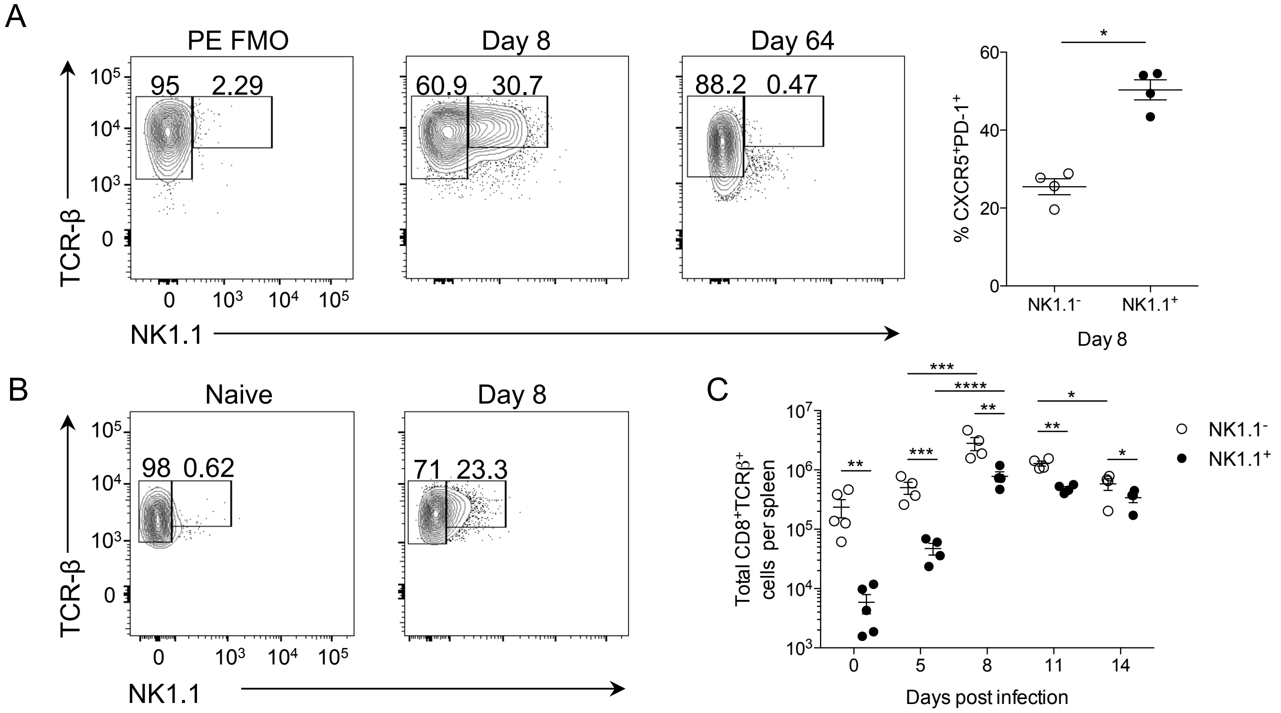

Supplemental Figure 1. NK1.1$^+$CD4$^+$ T cells expand during *P. chabaudi chabaudi* AS infection. (A) Representative TCRβ and NK1.1 expression at days 8 and 64 post-*P. c. chabaudi* AS infection. Cells previously gated on live CD4$^+$TCRβ$^+$ T cells. Frequency CXCR5$^+$PD-1$^+$ of NK1.1 positive or negative CD4$^+$TCRβ$^+$ T cells. Significance assessed by an unpaired nonparametric Mann-Whitney test. * $p < 0.05$. **(B)** Representative TCRβ and NK1.1 expression at day 8 post-*P. yoelii* infection. Cells previously gated on live activated (CD44$^+$CD62L$^-$) CD8$^+$TCRβ$^+$ T cells. **(C)** Total live NK1.1 positive or negative activated CD8$^+$TCRβ$^+$ T cells per spleen. An aligned rank transformation was performed prior to assessing the significance by two-way ANOVA with a post hoc Holm-Sidak's multiple comparisons test. * $p < 0.05$; ** $p < 0.01$; *** $p < 0.001$; **** $p < 0.0001$. Data are representative of two independent experiments (error bars, s.e.m.).
