# Supplementary material for: NK1.1 Expression Defines a Population of CD4+ Effector T Cells Displaying Th1 and Tfh Cell Properties That Support Early Antibody Production During Plasmodium yoelii Infection
Source: Front Immunol. 2018 Oct 15;9:2277. doi: 10.3389/fimmu.2018.02277 (PMC6196288; doi:10.3389/fimmu.2018.02277)
Supplement: Supplementary file 2 [file Data_Sheet_2.PDF]

Supplemental Figure 2

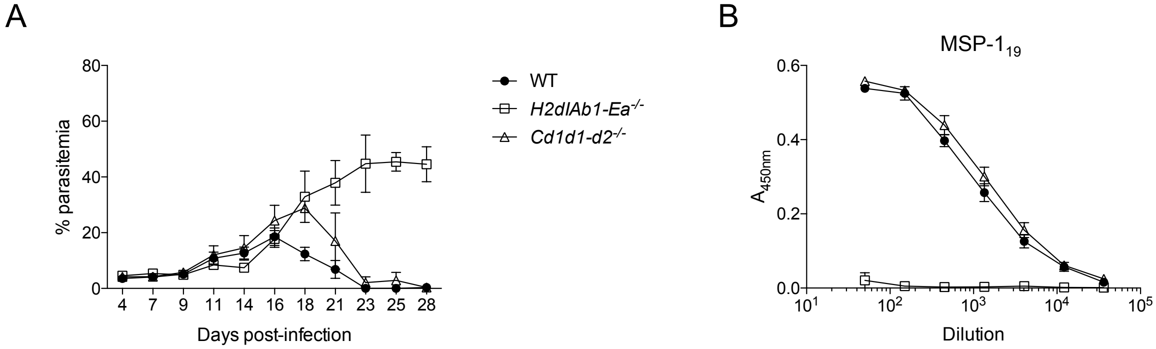

Supplemental Figure 2. CD1d-deficient mice have a higher peak parasitemia but display a similar capacity to produce antigen-specific class-switched Ab compared to WT mice. (A) Representative parasitemia curve for WT, MHC-II– and Cd1d–deficient mice infected with *P. yoelii*. **(B)** Measurement of MSP-1_{19}–specific IgG in total serum from WT, MHC-II– and Cd1d–deficient mice collected on day 28 p.i. Data are representative of three separate experiments (error bars, s.e.m.).
