# Supplementary material for: NK1.1 Expression Defines a Population of CD4+ Effector T Cells Displaying Th1 and Tfh Cell Properties That Support Early Antibody Production During Plasmodium yoelii Infection
Source: Front Immunol. 2018 Oct 15;9:2277. doi: 10.3389/fimmu.2018.02277 (PMC6196288; doi:10.3389/fimmu.2018.02277)
Supplement: Supplementary file 3 [file Data_Sheet_3.PDF]

Supplemental Figure 3

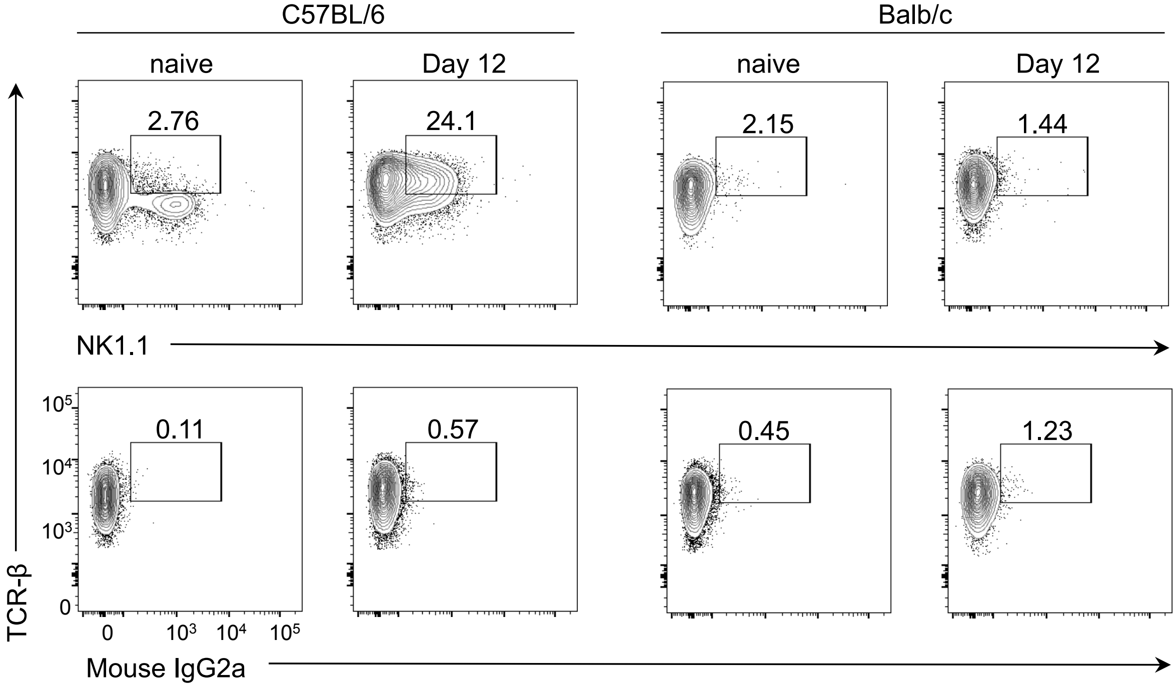

Supplemental Figure 3. Representative TCRβ and NK1.1 expression at day 12 post-infection in C57BL/6 and Balb/c mice. Mouse IgG2a, NK1.1-phycoerythrin isotype control. Data are representative of two independent experiments.
