# Supplementary material for: NK1.1 Expression Defines a Population of CD4+ Effector T Cells Displaying Th1 and Tfh Cell Properties That Support Early Antibody Production During Plasmodium yoelii Infection
Source: Front Immunol. 2018 Oct 15;9:2277. doi: 10.3389/fimmu.2018.02277 (PMC6196288; doi:10.3389/fimmu.2018.02277)
Supplement: Supplementary file 4 [file Data_Sheet_4.PDF]

Supplemental Figure 4

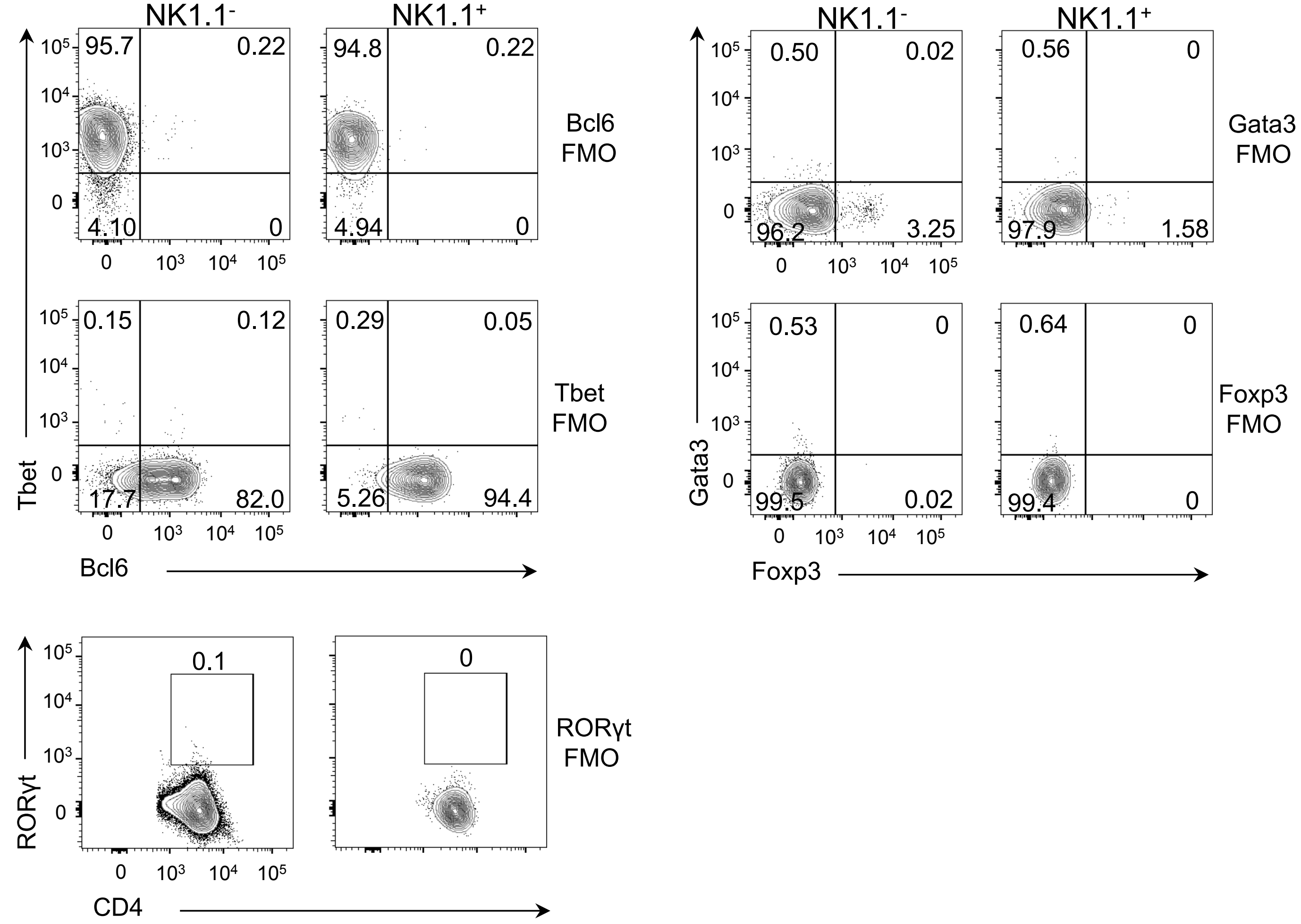

Supplemental Figure 4. Fluorescence minus one (FMO) controls for intracellular transcription factor staining. Gating strategy used to define cells positive and negative for expression of the $CD4^+$ T cell transcription factors Tbet, Bcl6, RORγt, Gata3 and Foxp3 within the $NK1.1^-$ and $NK1.1^+$ populations. Cells previously gated on live, $CD44^{hi}CD62L^{lo}CD4^+TCR\beta^+$ splenocytes.
