# Supplementary material for: NK1.1 Expression Defines a Population of CD4+ Effector T Cells Displaying Th1 and Tfh Cell Properties That Support Early Antibody Production During Plasmodium yoelii Infection
Source: Front Immunol. 2018 Oct 15;9:2277. doi: 10.3389/fimmu.2018.02277 (PMC6196288; doi:10.3389/fimmu.2018.02277)
Supplement: Supplementary file 5 [file Data_Sheet_5.PDF]

Supplemental Figure 5

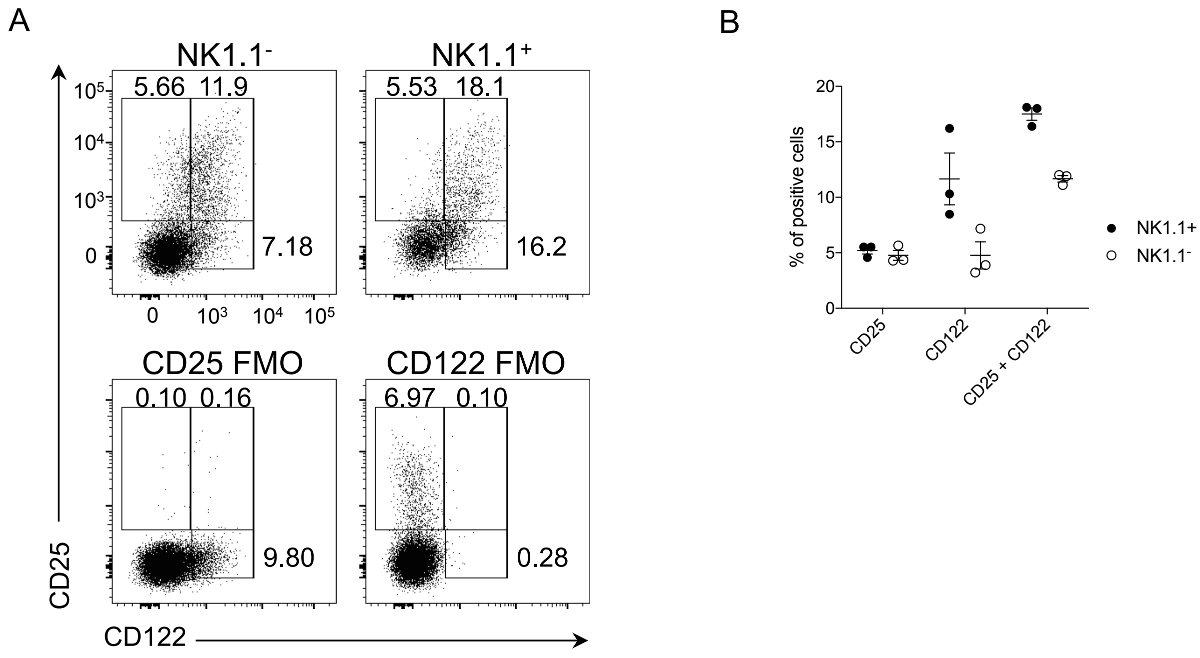

Supplemental Figure 5. A higher percentage of NK1.1$^+$ CD4$^+$ T cells express the IL-2Rα and –β chains at day 5 post-infection (A) Representative expression of IL-2Rα (CD25) and -β (CD122) chains within the NK1.1$^-$ and NK1.1$^+$ populations on day 5 p.i. Lower plots are representative fluorescence minus one (FMO) controls used to defined cells positive and negative for expression of CD25 and CD122. Cells previously gated on live, CD44^{hi}CD62L^{lo}CD4$^+$TCRβ$^+$ splenocytes. (B) Percentage of NK1.1$^-$ and NK1.1$^+$ CD4$^+$ T cells expressing CD25$^+$, CD122$^+$, and CD25$^+$CD122$^+$ on day 5 p.i. Data are representative of two independent experiments (error bars, s.e.m.).
